# Metabolic Anti-Cancer Effects of Melatonin: Clinically Relevant Prospects

**DOI:** 10.3390/cancers13123018

**Published:** 2021-06-16

**Authors:** Marek Samec, Alena Liskova, Lenka Koklesova, Kevin Zhai, Elizabeth Varghese, Samson Mathews Samuel, Miroslava Šudomová, Vincent Lucansky, Monika Kassayova, Martin Pec, Kamil Biringer, Aranka Brockmueller, Karol Kajo, Sherif T. S. Hassan, Mehdi Shakibaei, Olga Golubnitschaja, Dietrich Büsselberg, Peter Kubatka

**Affiliations:** 1 Clinic of Obstetrics and Gynecology, Jessenius Faculty of Medicine, Comenius University in Bratislava, 03601 Martin, Slovakia; marek.samec@uniba.sk (M.S.); liskova80@uniba.sk (A.L.); koklesova5@uniba.sk (L.K.); kamil.biringer@uniba.sk (K.B.); 2 Department of Physiology and Biophysics, Weill Cornell Medicine-Qatar, Education City, Qatar Foundation, Doha P.O. Box 24144, Qatar; kez4003@qatar-med.cornell.edu (K.Z.); elv2007@qatar-med.cornell.edu (E.V.); sms2016@qatar-med.cornell.edu (S.M.S.); 3 Museum of Literature in Moravia, Klašter 1, 66461 Rajhrad, Czech Republic; sudomova@post.cz; 4 Biomedical Centre Martin, Jessenius Faculty of Medicine in Martin, Comenius University in Bratislava, Mala Hora 4D, 036 01 Martin, Slovakia; vincent.lucansky@uniba.sk; 5 Department of Animal Physiology, Institute of Biology and Ecology, Faculty of Science, P. J. Šafarik University, 04001 Košice, Slovakia; monika.kassayova@upjs.sk; 6 Department of Medical Biology, Jessenius Faculty of Medicine, Comenius University in Bratislava, 03601 Martin, Slovakia; martin.pec@uniba.sk; 7 Musculoskeletal Research Group and Tumour Biology, Chair of Vegetative Anatomy, Institute of Anatomy, Faculty of Medicine, Ludwig-Maximilian-University Munich, D-80336 Munich, Germany; Aranka.Brockmueller@med.uni-muenchen.de (A.B.); mehdi.shakibaei@med.uni-muenchen.de (M.S.); 8 Department of Pathology, St. Elizabeth Cancer Institute Hospital, 81250 Bratislava, Slovakia; kkajo@ousa.sk; 9 Biomedical Research Centre, Slovak Academy of Sciences, 81439 Bratislava, Slovakia; 10 Department of Applied Ecology, Faculty of Environmental Sciences, Czech University of Life Sciences Prague, Kamýcká 129, 165 00 Prague, Czech Republic; sherif.hassan@seznam.cz; 11 European Association for Predictive, Preventive and Personalised Medicine, EPMA, 1160 Brussels, Belgium; olga.golubnitschaja@ukbonn.de; 12 Predictive, Preventive and Personalised (3P) Medicine, Department of Radiation Oncology, University Hospital Bonn, Rheinische Friedrich-Wilhelms-Universität Bonn, 53127 Bonn, Germany

**Keywords:** melatonin, cancer, metabolism, Warburg effect, mitochondrial dysfunction, anti-inflammatory, anti-depressant, antioxidant, anti-tumor, predictive preventive personalized medicine (PPPM/3PM)

## Abstract

**Simple Summary:**

Metabolic reprogramming is required for both malignant transformation and tumor development, including invasion and metastasis. Melatonin (5-methoxy-N-acetyltryptamine) is a methoxyindole that is synthesized in the pineal gland. Importantly, melatonin has anticancer effects by stimulating apoptosis, regulation of survival signaling, suppression of metastasis and angiogenesis and regulation of epigenetic modifications that contribute to malignant transformation. Furthermore, melatonin affects steps associated with the Warburg phenotype and suppresses the switch from oxidative phosphorylation to aerobic glycolysis through the regulation of critical enzymes and glucose transporters. Melatonin is involved in regulation of p53 and HIF-1, directly participate in signaling cascades that modulate aerobic glycolysis, gluconeogenesis, the tricarboxylic acid cycle and the pentose phosphate pathway. A significant impact of melatonin in the modulation of metabolic cascades represent a unique opportunity to inhibit pathways metabolic reprogramming.

**Abstract:**

Metabolic reprogramming characterized by alterations in nutrient uptake and critical molecular pathways associated with cancer cell metabolism represents a fundamental process of malignant transformation. Melatonin (N-acetyl-5-methoxytryptamine) is a hormone secreted by the pineal gland. Melatonin primarily regulates circadian rhythms but also exerts anti-inflammatory, anti-depressant, antioxidant and anti-tumor activities. Concerning cancer metabolism, melatonin displays significant anticancer effects via the regulation of key components of aerobic glycolysis, gluconeogenesis, the pentose phosphate pathway (PPP) and lipid metabolism. Melatonin treatment affects glucose transporter (GLUT) expression, glucose-6-phosphate dehydrogenase (G6PDH) activity, lactate production and other metabolic contributors. Moreover, melatonin modulates critical players in cancer development, such as HIF-1 and p53. Taken together, melatonin has notable anti-cancer effects at malignancy initiation, progression and metastasing. Further investigations of melatonin impacts relevant for cancer metabolism are expected to create innovative approaches supportive for the effective prevention and targeted therapy of cancers.

## 1. Introduction

Tumor cell metabolism is characteristically different from that of healthy cells [1]. The ability of cancer cells to modify their metabolism and adapt to nutrient-deprived environments to salvage nutrients and thus build biomass and accelerate proliferation is a well-known feature of malignant transformation [2]. Metabolic reprogramming of cancer cells is a hallmark of tumor development. Metabolic changes in tumor cells are driven by oncogenic mutations, hypoxic conditions, altered molecular signals that upregulate anabolic processes and the inhibition of catabolic cascades [1,3]. Changes in specific pathways, including glycolysis, gluconeogenesis, glutaminolysis, the pentose phosphate pathway (PPP), mitochondrial biogenesis and lipid metabolism, contribute to tumor development, invasion and metastasis [4]. Melatonin (C_13_H_16_N_2_O_2_; PubChem CID: 896; Available from: https://pubchem.ncbi.nlm.nih.gov/compound/Melatonin; (cited 20 April 2021)), a hormone secreted by the pineal gland, contributes to the regulation of circadian rhythms. Since its discovery (more than 60 years ago), melatonin has been extensively investigated in preclinical and clinical research [5]. Clinically, melatonin is used to manage sleep disorders, jetlag, depressive symptoms and anxiety [6,7]. Importantly, melatonin is a strong antioxidant and can protect organisms from carcinogenesis and neurodegeneration [7].

Moreover, melatonin has oncostatic effects by stimulating apoptosis, regulation of survival signaling, suppression of metastasis and angiogenesis and on the epigenetic machinery contributing to the malignant transformation demonstrated in vitro and in vivo [8,9,10,11]. Significantly, melatonin can attenuate the metabolic reprogramming of cancer cells [12,13]. Indeed, melatonin exerts a wide range of different effects, and its functional chemical groups play a key role in the induced oncostatic properties. This is illustrated by the chemical background, where melatonin belongs to the group of acetamides that is acetamide in which one of the hydrogens joined to the nitrogen atom is substituted by a 2-(5-methoxy-1H-indol-3-yl)ethyl group [14]. Acetamides have previously been reported to exhibit anticancer activities [15]. While experimental research has suggested a broad spectrum of melatonin’s anticancer abilities, the hormone’s impact on cancer metabolism requires further investigation. Understanding the processes behind melatonin’s effects on tumor metabolism can support the introduction of new therapeutic strategies to improve quality of life and prolong the overall survival of cancer patients in the context of preventive, predictive and personalized medicine.

### 1.1. Aim of the Study

This comprehensive review evaluates the effects of melatonin on cancer metabolism. Metabolic reprogramming of cancer cells promotes accelerated proliferation, acquisition of an invasive phenotype, metastasis and chemo/radio resistance development. The core of this manuscript focuses on melatonin’s role in the regulation of metabolic pathways in vitro and in vivo. Based on positive results from preclinical research, we emphasize a need to implement melatonin in the clinical sphere to attenuate metabolic transformations in tumor cells.

### 1.2. Source of the Data

Relevant data were collected from the biomedical literature using “melatonin“ and “cancer“ or “cancer metabolism“ or “aerobic glycolysis“ or “Warburg effect“ or “gluconeogenesis“ or “pentose phosphate pathway“ or “lipid metabolism“ or other associated terms as keywords or medical subject heading (MeSH) terms for searches in the PubMed database (https://pubmed.ncbi.nlm.nih.gov/ (data collected from January to April 2021)). We have focused on the recent publications from the years 2016–2021.

## 2. Structural and Functional Aspects of Melatonin

**Figure 1:**
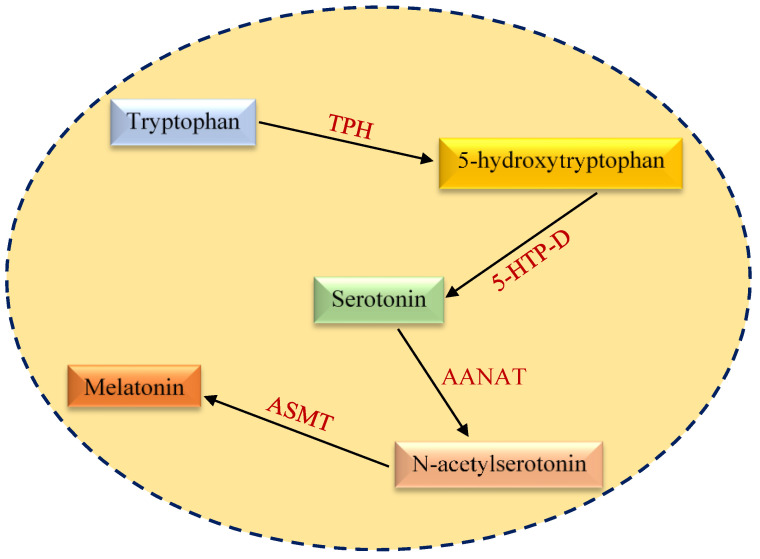
An overview of melatonin biosynthesis pathway. TPH, tryptophan hydroxylase; 5-HTP-D, 5-hydroxytryptophan decarboxylase; AANAT, arylalkylamine N-acetyltransferase; ASMT, acetylserotonin *O*-methyltransferase.

Melatonin, or 5-methoxy-N-acetyltryptamine, is a methoxyindole discovered in 1958 that is synthesized in the pineal gland (Figure 1) [16]. Melatonin is synthesized from tryptophan and secreted during the night (dark) phase of the day. Its secretion and synthesis are inhibited during the light phase of the day [7]. Tryptophan, an essential amino acid for melatonin synthesis, is hydroxylated into 5-hydroxytryptophan by tryptophan hydroxylase (TPH). 5-hydroxytryptophan is then converted to serotonin by 5-hydroxytryptophan decarboxylase [17]. Arylalkylamine N-acetyltransferase (AANAT) acetylates serotonin to form acetyl-serotonin and serves as a rate-limiting enzyme that regulates the rhythmic synthesis of melatonin [18,19]. Acetyl-serotonin is then converted to melatonin by acetylserotonin-*O*-methyltransferase (ASMT). Importantly, AANAT activity depends on cyclic AMP (cAMP) production. Light deficiency leads to norepinephrine release from sympathetic nerve fibers, resulting in cAMP synthesis [20]. Synthesized melatonin is released from the pineal gland into circulation [21]. Even though it is mainly produced in the pineal gland, melatonin is also produced elsewhere. Melatonin production occurs in various other tissues; however, these processes occur independently of circadian rhythms, and the synthesized melatonin is not released into circulation. Therefore, melatonin exists in two pools with different functions [7,22]. Melatonin regulates circadian rhythms, and the suprachiasmatic nucleus (SCN) regulates its circadian release. The SCN receives photic information about the environmental day/night cycle via the retinohypothalamic tract (RHT); melatonin biosynthesis occurs in the absence of light. Concurrently, melatonin controls SCN activity via feedback to its receptors (MT1 and MT2) in the SCN [23,24]. Dysregulation of melatonin-related pathways leads to sleep disorders and various health problems [25]. Moreover, melatonin exerts antioxidant and anti-inflammatory effects [26]. Its antioxidant role is associated with the neutralization of reactive nitrogen (RNS) and oxygen (ROS) species that affect the normal function of cells. Free radical accumulation due to disturbed oxidant-antioxidant machinery results in numerous pathological conditions [27,28]. Recent evidence suggests that melatonin treatment increases superoxide dismutase activity (SOD) and other antioxidant enzymes [29]. Moreover, melatonin stimulates an immune response through its receptors [30]. The immunoregulatory effects of melatonin are mediated by the stimulation of cytokines and acceleration of the T helper immune response. Melatonin promotes the production of interleukins (IL)-1, -6 and -12 by monocytes [31]. Moreover, melatonin supports antigen presentation by macrophages to T cells, resulting in cytotoxic T cell activation and proliferation [32]. Additionally, melatonin contributes to blood pressure regulation and autonomic control of cardiovascular function and has protective roles in various cardiovascular diseases [33]. Several studies reveal that melatonin inhibits carcinogenesis through various mechanisms [8,34,35,36,37]. Melatonin’s anticancer effects include pro-apoptotic [38], antiproliferative [39] and anti-angiogenic activities [10,40,41]. Moreover, melatonin exerts a tumor-suppressive capacity through the modulation of free radical scavenger action and immunoregulation via the activation of anticancer immune cells and the attenuation of T-regulatory cells (Tregs) and cancer-associated fibroblasts (CAF) [35,42].

### 2.1. Aberrations in Cancer Metabolism

Cancer cells are characterized by metabolic transformation, migration and uncontrolled proliferation [43]. Bioenergetic changes in tumors include the acceleration of glycolysis, elevation of glutaminolytic flux, enhancement of mitochondrial biogenesis, stimulation of the PPP and biosynthesis of macromolecules [4]. Alterations in glucose metabolism are essential features of cancer transformation; altogether, they constitute a shift from oxidative phosphorylation (OXPHOS) to glycolysis, even under normoxic conditions. This phenomenon is known as aerobic glycolysis or the Warburg effect [44,45]. Even though it is less energy efficient than OXPHOS (producing only 2 ATP molecules per glucose), aerobic glycolysis enables faster ATP production. Moreover, the metabolic switch from OXPHOS to aerobic glycolysis leads to the production of many intermediates, which are funneled into metabolic cascades for the generation of nucleotides, amino acids, NADPH and lipids [46]. Cellular glucose uptake is regulated by the functional glucose transporter (GLUT) family [47]. Increases in GLUT-mediated glucose uptake are characteristic of various tumor types, including breast cancer [48], prostate cancer [49], oral squamous cell carcinoma [50] and esophageal cancer [51]. Additionally, the transition from OXPHOS to the Warburg phenotype is promoted by the elevated expression of key glycolytic enzymes, such as hexokinase 2 (HK2), phosphofructokinase-1 (PFK1), lactate dehydrogenase A (LDHA) and pyruvate kinase type M2 (PKM2), which are associated with neoplastic transformation [52,53,54,55]. Changes in molecular cascades such as the phosphoinositide 3-kinase/Akt/mammalian target of rapamycin (PI3K/Akt/mTOR) pathway, upregulation of hypoxia-inducible factor 1 (HIF-1) and c-MYC, insufficient p53-mediated control and epigenetic mechanisms all contribute to the deregulation of glycolytic enzymes [56,57].

Gluconeogenesis is responsible for generating glucose from non-carbohydrate carbon precursors such as pyruvate, lactate, propionate and glycerol [58,59]. Seven enzymes are shared between glycolysis and gluconeogenesis. Furthermore, there are four enzymes unique to gluconeogenesis: pyruvate carboxylase, which converts pyruvate to oxaloacetate (OAA); phosphoenolpyruvate carboxykinase (PCK), which catalyzes the conversion of OAA to phosphoenolpyruvate (PEP); fructose-1,6-bisphosphatase (FBPase), which converts fructose 1,6-bisphosphate (F1,6P) to fructose 6-phosphate (F6P); and glucose-6-phosphatase (G6Pase), which hydrolyzes glucose-6-phosphate [58]. In cancer cells, gluconeogenesis generates intermediate metabolites necessary for biomolecule synthesis, especially during glucose deprivation [58]. Moreover, the key enzymes of gluconeogenesis (PCK, FBPase, G6Pase) affect cell survival, signaling and proliferation, as well as cancer stem cell (CSC) phenotypes [60]. Several studies revealed that cytoplasmatic PCK1 and mitochondrial PCK2 contribute to cancer growth [61,62,63]. Additionally, FBP1 deficiency was documented in lung [64], breast [65] and renal cancers [66].

The tricarboxylic acid (TCA) cycle is a central hub of oxidative metabolism, synthesis of macromolecules and redox balance [67]. The TCA cycle is a series of enzyme-catalyzed biochemical reactions [68]. Deficiencies in succinate dehydrogenase (SDH), fumarate hydratase (FH) and isocitrate dehydrogenase (IDH) due to mutations (inherited or acquired) result in metabolic changes [69]. The accumulation of succinate and fumarate due to defects in SDH and FH leads to the inhibition of prolyl hydroxylase enzymes (PHD), stabilization of HIF-1α and subsequent acceleration of glycolysis in cancer cells [70]. In contrast, the accumulation of α-ketoglutarate results in the destabilization of HIF-1α [71]. Moreover, alterations of c-MYC, P53 or RAS may modulate the TCA cycle [72].

The PPP is an important cascade in glucose metabolism [73]. The PPP is responsible for the generation of the reduced form of nicotinamide adenine dinucleotide phosphate (NADPH) and the production of ribose 5-phosphate (R5P) that is necessary for nucleotide synthesis [74]. Glucose-6-phosphate (G6P) is a major precursor that enters the PPP from glycolysis. The PPP consists of an oxidative and a non-oxidative phase [75]. Glucose-6-phosphate dehydrogenase (G6PDH), 6-phosphogluconolactonase (6PGL) and 6-phosphogluconate dehydrogenase (6PGDH) are crucial for the synthesis of ribulose-5 phosphate and NADPH through the oxidative phase [76]. The non-oxidative phase is characterized by a series of non-oxidative reactions leading to the synthesis of five-carbon sugars that serve as precursors for nucleotide biosynthesis or glycolytic intermediates (e.g., F6P and glyceraldehyde-3-phosphate (G3P)) [77,78]. PPP deregulation is frequently observed during cancer development to fulfill the increased R5P and NADPH requirements of rapidly dividing cancer cells [75]. G6PDH is upregulated in renal cell [79], breast [80], gastric [81] and colon cancers [82]. Similarly, the expression levels of other PPP enzymes (6PGL, 6PGDH) change during cancer development [73,83,84]. Molecular analyses of PPP regulation revealed several mechanisms; deregulation of these mechanisms lead to a cancerous phenotype. Alterations in p53 activity increased glucose uptake in tumor cells through the upregulation of GLUT1 and GLUT4; this led to elevated G6P levels for the PPP and glycolysis [85,86]. Moreover, alterations in mTOR [87], nuclear factor erythroid 2-related factor 2 (Nrf2) [88] and KRAS [89] were associated with modulation of the PPP.

Metabolic reprogramming is common to many cancer types and is suggested to play a significant role in developing therapeutic resistance [90]. Moreover, monitoring for areas of high glucose uptake is utilized for cancer diagnosis and treatment. It is, therefore, logical that key elements of the enzymatic cascades involved in aerobic glycolysis are considered potential targets for anticancer therapies [91]. Beyond glucose metabolism, the metabolism of amino acids and lipids is altered significantly by cancer development and progression; thus, there are many possibilities for interference with pathologically modified pathways [92,93]. Nonetheless, only a few anticancer agents modulating metabolism are clinically available [94].

### 2.2. Links between Mitochondrial Dysfunction, Melatonin and Cancer

Recent evidence revealed differences in tumor metabolisms during the daytime and nighttime. During the day, cancer cells manifest the Warburg phenotype associated with an elevated level of cytosolic glycolysis. On the other hand, at night tumors exhibit decreased aerobic glycolysis and metabolic reprogramming leading to OXPHOS, and thus cancer cells use mitochondrial oxidation of glucose to ATP generation [95]. Acquired data showed that metabolic reprogramming of cancer cells to healthy phenotype correlated with rising of circulating melatonin during the night. Importantly, tumor-bearing animals exposed to light during the night exhibited inhibition of nocturnal melatonin, resulting in increased glucose uptake and lactate secretion [96]. As was discussed previously, glucose is transported into cells via glucose transporters. In healthy cells, glucose is converted to pyruvate that enters to mitochondria, and subsequently, pyruvate is transformed to acetyl CoA via pyruvate dehydrogenase complex (PDC) [97]. Acetyl CoA plays an essential role in the delivery of the acetyl group to the TCA. Cancer cells are characterized by disruption of acetyl CoA synthesis due to upregulation of pyruvate dehydrogenase kinase (PDK), directly inhibiting PDC. An anticancer drug such as DCA can inhibit PDK [98]. Similar to DCA, blood circulating melatonin secreted by pineal glands is presumably able to inhibit PDK activity, reactivates PDC and thus reverses the Warburg effect [99]. Prediction of melatonin’s role in PDK inhibition is associated with the ability of melatonin to enter mitochondria. Based on recent studies, it has been observed that peripherally injected melatonin accumulates in mitochondria [100].

It is generally known that mitochondria of healthy cells produce melatonin. Intramitochondrial melatonin could suppresses PDK activity and blocks metabolic reprogramming leading to aerobic glycolysis. The assumed deprivation of melatonin in cancer cells, particularly during the day, is associated with the interrupted biogenesis of melatonin in mitochondria. Acetyl CoA plays an important role in melatonin synthesis. Acetyl CoA is the necessary substrate for the rate-limiting enzyme AANAT that contributes to the melatonin synthetic pathway [18,101]. An insufficient amount of acetyl CoA in cancer cell mitochondria disrupt melatonin synthesis, so these cells cannot produce their own melatonin. An elevated level of circulating melatonin by the pineal gland during the night would suppress PDK activity and allowing tumor cells metabolic switch from aerobic glycolysis to mitochondrial OXPHOS [102].

Only more in-depth analyzes of mechanisms by which melatonin affects mitochondrial oxidative phosphorylation can bring novel therapeutic strategies for effective inhibition of metabolic reprogramming of cancer cells.

### 2.3. Melatonin Regulating Cancer Metabolism In Vitro

As mentioned, bioenergetic alterations are required for malignant transformation and tumor progression. New molecular insights beyond metabolic reprogramming introduce new opportunities to target essential steps of cancer-associated energetic processes. Melatonin affects steps associated with the Warburg phenotype and suppresses the switch from OXPHOS to aerobic glycolysis [12].

Impressive effects of melatonin on glucose metabolism were observed in two prostate cancer (androgen-sensitive LNCaP and insensitive PC-3) cell lines using a 13C stable isotope. Acquired data revealed that melatonin reduced glucose uptake in prostate cancer cells. Moreover, melatonin in the culture medium significantly reduced the ATP/AMP ratio and lactate 13C-labeling in both androgen-sensitive and androgen insensitive cancer cells and downregulated the TCA in LNCaP cells. Furthermore, lactate dehydrogenase (LDH) activity was reduced in LNCaP cells incubated in a melatonin-containing medium. G6PDH is another enzyme of the PPP. Indeed, PPP activity (measured by an increment of G6PDH) was significantly reduced in androgen-sensitive LNCaP cells cultured in a melatonin-containing glucose medium [103]. Antitumoral effects of melatonin were also documented in Ewing sarcoma (TC-71, A-673 and A-4573) and chondrosarcoma (sw-1353) cells. Ewing sarcoma cells exhibit metabolically reprogrammed phenotypes, as observed through elevated LDH activity, increased glucose uptake and activated HIF-1α. On the other hand, chondrosarcoma cells do not exhibit the Warburg phenotype. Decreased glucose uptake was observed in all three Ewing sarcoma cell lines cultured with melatonin, but no changes were observed in sw-1353 cells. Similarly, LDH activity was reduced in TC-71, A-673 and A-4573 cells, while no changes in lactate level or LDH activity were documented in sw-1353 chondrosarcoma cells. Interestingly, melatonin exerts regulatory effects on HIF-1α, as evidenced by the accumulation of inactive (hydroxylated) HIF-1α in TC-71, A-673 and A-4573 cells following melatonin treatment [104]. Recent evidence suggested a potent antineoplastic effect of melatonin by regulating cisplatin resistance and glucose metabolism mediated by Hippo signaling in hepatocellular carcinoma (HCC) HepG2 and Hep3B cells. The Hippo signaling pathway regulates tissue growth, and its deregulation is associated with tumorigenesis [105]. Moreover, the Yes-associated protein (YAP) is a downstream effector of the Hippo; its ectopic expression induces oncogenic transformation [106,107]. Melatonin treatment suppressed cancer metabolism in HCC HepG2 and Hep3B cells by downregulating GLUT3 transporters and consequently inhibiting glucose uptake and ATP production. Concerning the apoptotic cascade, melatonin downregulated Bcl-2 in both HCC cell lines. Lower mRNA and protein levels of YAP were further identified as consequences of melatonin intervention. On the other hand, higher levels of YAP were detected in HepG2 and Hep3B cells without melatonin treatment; these partially reversed the melatonin-supported suppression of proliferation, metabolic reprogramming and apoptosis mediated by cisplatin [108]. Furthermore, recent evidence showed the regulatory effects of melatonin on nickel-induced metabolic changes. Nickel induces carcinogenesis and promotes the Warburg phenotype through the stabilization of HIF-1α due to ROS generation. As described above, melatonin directly increases cellular ROS scavenging [109]. Thus, melatonin attenuated the nickel-mediated metabolic switch from OXPHOS to aerobic glycolysis in a normal bronchial epithelium (BEAS-2B) cell line. Further analysis revealed that melatonin suppressed molecular components of aerobic glycolysis, such as miR-210, iron-sulfur cluster assembly scaffold protein (ISCU1/2) and HIF-1α [110]. Moreover, FMS-like tyrosine kinase 3 (FLT3) internal tandem duplication (ITD) is the most common genetic alteration observed in patients with acute myeloid leukemia [111]. Recent evidence points to a significant role of FLT3-ITD in cancer cell proliferation, survival and metabolic reprogramming. Puente-Moncada et al. [112] evaluated differences in proliferation, apoptosis and glucose metabolism between AML cell lines with (MV-4-11 and MOLM-13) and without FLT3-ITD mutations (OCI-AML3 and U-937) after melatonin treatment. Melatonin induced apoptosis in MV-4-11 and MOLM-13 cells, but only suppressed the proliferation of OCI-AML3 and U-937 cells. In addition, melatonin inhibited tumor growth and prolonged overall survival in FT3-ID AML xenografts. Analysis of metabolic changes showed that melatonin decreased glucose uptake, LDH activity and lactate generation, and HIF-1α activation [112]. Last but not least, melatonin affected cancer metabolism in Cal-27 and SCC-9 head and neck squamous cell carcinoma (HNCC) cells by regulating mitochondrial function and structure. Melatonin stimulated OXPHOS and suppressed cytosolic aerobic glycolysis leading to ROS production, mitophagy, apoptosis and reduction of cell proliferation [113].

### 2.4. Impact of Melatonin on the Metabolic Reprogramming In Vivo

Up to now, several studies have investigated the impact of melatonin on metabolic changes in animal models of carcinogenesis. Recent evidence revealed that melatonin is transported into cells by glucose transporters. This process reduced glucose uptake in prostate cancer cells and inhibited glucose-induced tumor growth and proliferation in a model of transgenic adenocarcinoma of the mouse prostate (TRAMP) [114]. Moreover, Dauchy et al. [115] evaluated the connections between light intensity, duration, spectral quality, melatonin and cancer metabolism. The authors tested the hypothesis that blue light (white light through blue-tinted cages) during the day amplifies nocturnal melatonin and result in the suppression of metabolism and growth in prostate cancer xenografts. Interestingly, acquired data suggested that the Warburg effect (lactate production and glucose uptake) and tumor growth were inhibited in PC3 xenografts in blue cages compared to clear cages. Based on these results, daytime blue light exposure can affect the circadian reorganization of metabolic and hormonal processes, thereby inhibiting cancer metabolism, growth and proliferation [115]. Additionally, nighttime light exposure disrupted the circadian organization in a breast cancer MCF-7 xenograft model. Dim light at night inhibited melatonin release from the pineal gland. The deregulation of the circadian clock affected breast cancer progression by enhancing aerobic glycolysis, proliferation and lipid signaling [96]. Moreover, melatonin affected glucose metabolism and doxorubicin resistance in MCF-7 xenografts in nude rats. Dim light exposure at night disrupted circadian stimulation by melatonin in the tested animals. Melatonin intervention affected metabolic reprogramming by decreasing glucose uptake and lactate release. Furthermore, melatonin re-established the doxorubicin sensitivity of breast cancer cells [116]. Similarly, dim light exposure suppressed melatonin release in female athymic inbred nude rats. Experimental findings showed that the disruption of nocturnal melatonin release promoted tamoxifen resistance and stimulated metabolic reprogramming associated with tumor growth and proliferation [117]. In addition, melatonin affected metabolic pathways in 7,12-dimethylbenz(a)anthracene (DMBA)-induced ovarian carcinogenesis in vivo by downregulating key proteins that regulate HIF-1 signaling, energy generation and the production of metabolites essential for tumor growth and proliferation [118]. Likewise, in a study focusing on leiomyosarcoma, melatonin suppressed the Warburg phenotype in SK-LMS-1 xenografts. Indeed, melatonin intervention inhibited glucose uptake and lactate production in vivo [119].

In conclusion, melatonin suppresses the metabolic reprogramming of cancer cells by modulating various signaling cascades, including those related to glucose transporters and key glycolytic enzymes. Table 1 summarizes recent experimental in vitro and in vivo studies investigating the regulatory effects of melatonin on metabolic changes associated with cancer development.

### 2.5. Melatonin’s Impact on the “Critical Players” of Metabolic Reprogramming

As discussed earlier, several critical players contribute to the metabolic reprogramming of cancer cells. Among them, p53 and HIF-1 directly participate in signaling cascades that modulate aerobic glycolysis, gluconeogenesis, the TCA cycle and the PPP [120,121,122,123,124]. Several experimental studies analyzed melatonin’s impact on these critical regulators. Melatonin promoted apoptosis and cell cycle arrest in HCC HepG2 cells by activating caspase-3, -8 and -9, BAX, poly (ADP-ribose) polymerase (PARP) proteolysis and cytochrome C release. Notably, p53 was upregulated in the tested HepG2 cell line [125]. Furthermore, melatonin significantly upregulated p53 and caspase-3 and -9, and downregulated Bcl-2 at the mRNA level, in Ehrlich ascites carcinoma cells inoculated into BALB/c mice; this inhibited tumor growth, proliferation and neovascularization [126]. Interestingly, melatonin also exerts anticancer abilities in combination with phytochemicals. Naturally occurring phytochemicals have numerous beneficial effects on human health [127,128,129]. They exhibit anticancer efficacy through the regulation of molecular cascades associated with cancer initiation, promotion and progression, including those for angiogenesis [130,131,132,133], hypoxia [134], metabolism [44], metastasis [135], apoptosis [136,137] and epigenetic machinery [138,139,140]. Zhang et al. [141] evaluated the combined anticancer effects of melatonin and epigallocatechin-3 gallate (EGCG) in the HCC HepG2 cell line. Melatonin suppressed p21 and thereby sensitized HepG2 cells to EGCG toxicity. Indeed, lower expression of p21 is correlated with the melatonin-induced downregulation of p53 in HepG2 cells. Experimental data indicated that EGCG and melatonin are together more effective against cancer cells, as melatonin both reduces EGCG hepatotoxicity and increases EGCG’s anticancer capacity [141]. Nowadays, excessive intake of phytochemicals is connected to side effects such as hemolytic anemia and hepatotoxicity [142]. However, melatonin exerts protective effects against phytochemical-induced side effects, as demonstrated through its attenuation of high-dose EGCG-induced hepatotoxicity in vivo [143]. Moreover, activation of the PI3k/Akt/Mouse double minute 2 homolog (MDM2) pathway induces metabolic reprogramming due to increased p53 degradation [144]. Nevertheless, melatonin blocked the Akt/MDM2 pathway and subsequently upregulated p53, leading to the inhibition of proliferation and induction of apoptosis in gastric cancer SGC-7901 cells [145]. Similarly, melatonin suppressed MDM2 gene expression and blocked its nuclear transport in breast cancer MCF-7 cells. On the other hand, melatonin significantly increased p53 acetylation to protect it from MDM2-dependent degradation, increasing p53 activity in the same MCF-7 cell line [146].

Moreover, HIF-1 plays an essential role in metabolic reprogramming by regulating enzymes associated with the Warburg phenotype. As the adaptive response to hypoxia, HIF-1 represents a promising target in anticancer therapy [122,134]. Indeed, melatonin can affect cancer metabolism through the regulation of HIF-1. Melatonin downregulated HIF-1α and vascular endothelial growth factor (VEGF) in human pancreatic cancer (PANC-1), cervical cancer (HeLa) and lung adenocarcinoma (A549) cell lines under hypoxic conditions mimicked by cobalt chloride [147]. An analogous phenomenon was documented in human umbilical vein endothelial cells (HUVECs) under hypoxic and normoxic conditions, as melatonin inhibited the HIF-1/ROS/VEGF cascade [41]. Furthermore, in mice inoculated with BALB/c-derived renal adenocarcinoma cells (RENCA), melatonin suppressed tumor growth and neovascularization by reducing HIF-1 activity [148]. Moreover, the invasive properties of the HCC HepG2 cell line were disrupted by melatonin, which suppressed proliferation and neovascularization by downregulating VEGF and HIF-1α [149]. In addition, downregulation of HIF-1α was observed in prostate cancer DU145, PC-3 and LNCaP cell lines after melatonin treatment. Indeed, the downregulation of HIF-1α was mediated by the dephosphorylation of p70S6K and RPS6, which regulate HIF-1α expression at the translational level [150]. Finally, melatonin antagonized hypoxia-mediated migration and invasion by suppressing HIF-1α, matrix metalloproteinase 2 (MMP2) and VEGF in glioblastoma U251 and U87 cells [151].

**Figure 2:**
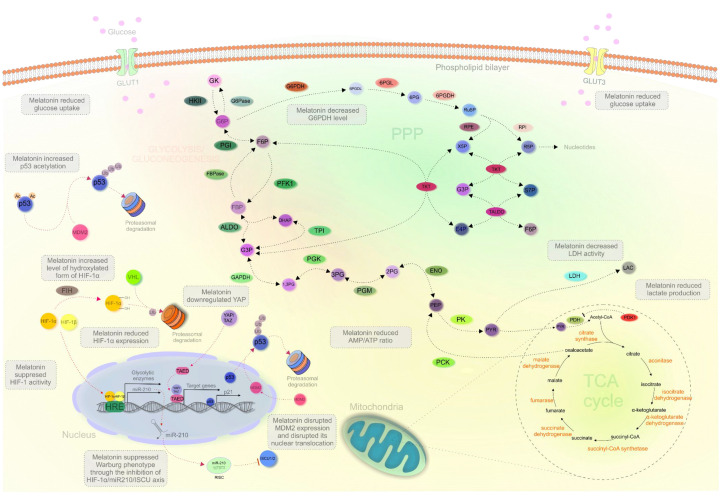
Melatonin targeting cancer metabolism. Abbreviations: GK, glucose; HKII, hexokinase II; G6P, glucose 6-phosphate; F6P, fructose 6-phosphate; PGI, phosphoglucose isomerase; PFK1, phosphofructokinase 1; FBP, fructose-1,6-bisphosphate; DHAP, dihydroxyacetone phosphate; G3P, glyceraldehyde 3-phosphate; 1,3PG, 1,3-bisphosphoglycerate; 3PG, 3-phosphoglycerate; 2PG, 2-phosphoglycerate; PEP, phosphoenolpyruvate; PYR, pyruvate; LAC, lactate; ALDO, aldolase; TPI, triosephosphate isomerase; GAPDH, glyceraldehyde 3-phosphate dehydrogenase; PGK, phosphoglycerate kinase; PGM, phosphoglycerate mutase; ENO, enolase; PK, pyruvate kinase; LDH, lactate dehydrogenase; PDH, pyruvate dehydrogenase; PDK1, pyruvate dehydrogenase kinase 1; PCK, phosphoenolpyruvate carboxykinase; FBPase, fructose-1,6-bisphosphatase; G6Pase, glucose-6-phosphatase; G6PDH, glucose-6-phosphate dehydrogenase; 6PGDL, 6-phosphogluconolactone; 6PG, 6-phosphogluconate; Ru5P, ribulose 5-phosphate; X5P, xylulose 5-phosphate; R5P, ribose 5-phosphate; S7P, sedoheptulose 7-phosphate; E4P, erythrose 4-phosphate; TKT, transketolase; TALDO, transaldolase; RPE, ribulose-5-phosphate 3-epimerase; RPI, ribose-5-phosphate isomerase; 6PGDH, 6-phosphogluconate dehydrogenase; 6PGL, 6-phosphogluconolactonase; GLUT1/3, glucose transporter 1/3; MDM2, mouse double minute 2; Ac, acetylation; Ub, ubiquitination; HIF-1α/1β, hypoxia-inducible factor 1α/1β; VHL, von Hippel–Lindau; FIH, factor inhibiting HIF; YAP/TAZ, yes-associated protein/WW domain–containing transcription regulator 1; HRE, HRE, hypoxia-response elements; TAED, transcriptional enhancer factor TEF-1; RISC, RNA-induced silencing complex; ISCU 1/2, iron-sulfur cluster assembly scaffold protein ½.

In conclusion, melatonin can potently suppress the Warburg phenotype by modulating p53 and HIF-1. An overview of melatonin’s anticancer properties is provided in Table 2. Figure 2 provides a complex overview of melatonin´s impact on cancer metabolism, as described in this review.

## 3. Expert Recommendations in the Framework of Predictive, Preventive and Personalized (3P) Medicine

Melatonin demonstrates protective effects in sleep disturbances and sleep-related disorders, depression, mitochondrial dysfunction, chronic inflammation as well as their interrelationship highly relevant for carcinogenesis and other related pathologies such as stroke [6,25,45,152,153,154,155,156,157,158]. Based on current experimental research, melatonin is proposed to be used as the pharmacological agents with a multi-functional capacity such as to modulate mitochondrial functions in cancer, among others [159]. Current preclinical evidence suggests that melatonin can modulate different molecular cascades directly connected to the suppression of cancer development and progression. Antitumor efficacy of melatonin mediated by inhibition of metastatic potential was demonstrated in different in vitro model systems [160,161,162]. Even though a number of experimental studies confirmed anticancer abilities of melatonin, massive clinical testing is still absent. The imminent need to identify and investigate novel therapeutic approaches in cancer research determines melatonin as a promising agent targeted on cancer combined with conventional therapies. Further investigation on melatonin’s role in cancer initiation and progression can improve its therapeutical potential in the clinical sphere [102,163,164,165].

The above discussed preclinical research provides valuable insights into the effects of melatonin on tumor metabolism and the Warburg phenotype, an essential step for unrestricted tumor cell proliferation and cancer progression. Melatonin regulates the critical components associated with cancer metabolism, such as GLUTs, LDH, G6PDH, HIF-1 or p53. [96,103,104,108,110,112,114,115,116,117,118,119]. Following notable preclinical achievements in the field, we emphasize the necessity to investigate melatonin effects focused on the clinical needs [166,167,168,169].

To this end, population screening focused on individuals in sub-optimal health conditions prior to the clinical onset of the pathologies followed by cost-effective targeted treatment is considered the optimal approach in the framework of 3P medicine as a concept of medicine of the 21st century [170,171,172].

## 4. Conclusions

In conclusion, comprehensive knowledge of melatonin’s capacity to regulate tumor metabolism are expected to strongly contribute to the identification of innovative approaches to an improved cancer management. The above-discussed results of preclinical research provide valuable knowledge about the effect of melatonin on tumor metabolism and the Warburg phenotype, an essential step for unrestricted tumor cell proliferation and cancer progression. Despite significant results from preclinical research, we emphasize the need to further investigate the effect of melatonin on cancer processes through the regulation of the Warburg phenotype within the scope of other more complex modalities of cancer research, including clinical investigation.

## Figures and Tables

**Table 1:** Melatonin targeting cancer metabolism in preclinical research.

Study Design	Effects of Melatonin	Mechanism	Reference
In Vitro			
PC3, LNCaP cells	↓ Glycolysis ↓ TCA ↓ PPP	Reduced the ATP/AMP ratio, lactate labeling, LDH activity and G6PDH	[103]
TC-71, A-673 and A-4573 cells	↓ Glycolysis	Inhibited LDH activity; reduced glucose uptake; upregulated the hydroxylated (inactivated) form of HIF-1α	[104]
HepG2, Hep3B cells	↓ Glucose uptake ↓ Resistance to cisplatin ↓ Proliferation	Downregulated YAP and subsequently regulated the Hippo signaling pathway; suppressed Bcl-2 and GLUT3 expression	[108]
BEAS-2B cells	↓ Glycolysis	Melatonin suppressed the nickel-induced Warburg effect by inhibiting the HIF-1α/miR210/ISCU axis	[110]
MV-4-11, MOLM-13, OCI-AML3, U-937 cells	↓ Glycolysis ↓ Proliferation ↑ Apoptosis	Melatonin regulated glucose metabolism by attenuating glucose uptake, LDH activity, lactate secretion and HIF-1α activation	[112]
Cal-27, SCC-9 cells	↓ Glycolysis ↓ Proliferation ↑ Apoptosis ↑ Mitophagy ↑ ROS production	Melatonin intervention increased the level of acetyl CoA succinyl CoA, citric acid, NADH and reduced the level of pyruvate. Melatonin also increased OXPHOS level leading to suppression of aerobic glycolysis. Melatonin stimulated mitochondrial function, resulting in oxidative stress and subsequent apoptosis and mitophagy	[113]
In Vivo			
PC3, LNCaP cells; TRAMP mice	↓ Glucose uptake ↓ Tumor progression	Reduced glucose uptake via GLUT1 in prostate cancer cells; inhibited glucose-induced tumor progression in mice	[114]
PC3 xenografts in male nude rats	↓ Glycolysis ↓ Proliferation ↓ Growth	Daytime blue light exposure amplified supraphysiologic nocturnal melatonin release, resulting in the suppression of cancer progression	[115]
MCF-7 xenografts in Weanling female nude rats	↓ Glycolysis ↓ Lipid signaling ↓ Proliferation	Dim light at night disrupted the circadian cycle associated with melatonin release, leading to the promotion of cancer processes such as aerobic glycolysis, proliferation and lipid signaling	[96]
MCF-7 xenografts in female nude rats	↓ Glycolysis ↑ Sensitivity to doxorubicin	Disruption of the circadian release of melatonin due to dim light exposure at night affected cancer metabolism and doxorubicin resistance. Conversely, melatonin suppressed lactate release and glucose uptake and restored the sensitivity of cancer cells to doxorubicin	[116]
MCF-7 xenografts in female nude rats	↑ Sensitivity to tamoxifen ↓ Glycolysis	Disruption of melatonin release due to dim light exposure at night led to tamoxifen resistance and enhanced cancer metabolism. These characteristics were not identified in animals without circadian rhythm disruption or after supplementation with melatonin	[117]
DMBA-induced ovarian carcinogenesis in Fisher 344 rats	↓ Proteins associated with cancer metabolism pathways	Melatonin administration for 60 days decreased the levels of proteins related to metabolic cascades, including proteins contributing to mitochondrial systems, HIF-1 signaling and generation of metabolites	[118]
SK-LMS-1 xenografts	↓ Glycolysis	Melatonin suppressed the Warburg effect by decreasing glucose uptake and lactate production	[119]

Explanatory notes: ↓ decrease, reduction, suppression; ↑ induction, increase. Abbreviations: HIF-1, hypoxia-inducible factor 1; HIF-1α, hypoxia-inducible factor 1α; ISCU, iron-sulfur cluster assembly scaffold protein; GLUT1/3, glucose transporter 1/3; YAP, yes-associated protein; LDH, lactate dehydrogenase; G6PDH, glucose-6-phosphate dehydrogenase.

**Table 2:** Melatonin’s impact on critical regulators related to cancer metabolism.

Study Design	Effects	Mechanism	Reference
HepG2 cells	↑ Apoptosis → Cell cycle arrest	Melatonin exhibited oncostatic abilities through the upregulation of caspase-3, -8 and -9, p53 and Bax; cytochrome C release; and the activation of Poly (ADP-ribose) polymerase (PARP) proteolysis	[125]
Ehrlich ascites carcinoma cells inoculated into BALB/c mice	↓ Growth ↓ Proliferation ↓ Angiogenesis	Melatonin downregulated Bcl-2 and upregulated p53 and caspase-3 and -9	[126]
HepG2 cells	↑ Sensitization of cancer cells to EGCG toxicity ↓ Risk of EGCG-induced hepatoxicity	Melatonin downregulated p21 and subsequently sensitized cancer cells to EGCG toxicity	[141]
SGC-7901 cells	↓ Proliferation ↑ Apoptosis	Melatonin blocked the Akt/MDM2 cascade, resulting in p53 activation	[145]
MCF-7 cells	↓ Growth ↑ Apoptosis	Melatonin inhibited MDM2 expression and disrupted MDM2 nuclear translocation Melatonin induced p53 activation and increased p53 acetylation/stabilization	[146]
PANC-1, HeLa and A549 cells	↓ Angiogenesis	Melatonin decreased VEGF and HIF-1α in cancer cells	[147]
HUVEC	↓ Angiogenesis under hypoxia	Melatonin suppressed the HIF-1/ROS/VEGF cascade	[41]
RENCA cells inoculated into BALB/c mice	↓ Growth ↓ Angiogenesis	Melatonin reduced HIF-1 activity in the animal model	[148]
HepG2 cells	↓ Invasiveness ↓ Proliferation ↓ Angiogenesis	Melatonin downregulated HIF-1α and VEGF	[149]
DU145, PC-3 and LNCaP cells	↓ Angiogenesis	Melatonin inhibited HIF-1α protein synthesis through the dephosphorylation of p70S6K and RPS6	[150]
U251 and U87 cells	↓ Migration ↓ Invasion	Melatonin downregulated HIF-1α, MMP2 and VEGF	[151]

Explanatory notes: ↓ decrease, reduction, suppression; ↑ induction, increase; → induction. Abbreviations: HIF-1, hypoxia-inducible factor 1; HIF-1α, hypoxia-inducible factor 1α; VEGF, vascular endothelial growth factor; MMP2, matrix metalloproteinase 2; MDM2, mouse double minute 2 homolog; ROS, reactive oxygen species.

## Data Availability

Data is contained within the article.
